# Opthalmia, Ulceration of the Cornea, Being the Substance of a Clinical Lecture to the Class Attending the Mercy Hospital; Delivered in Jan. 1857

**Published:** 1857-06

**Authors:** N. S. Davis

**Affiliations:** Prof. of Prin. and Practice of Med. and Clin. Med.


ARTICLE IV.—*Opthalmia, Ulceration of the Cornea, being the Substance of a Clinical Lecture to the class attending the Mercy Hospital, delivered in Jan.* 1857. By N. S. DAVIS, M.D., Prof. of Prin. and Practice of Med. and Clin. Med.

*Gentlemen*—I shall direct your attention this morning chiefly to some cases of disease of the eye. For though this class of diseases is generally included in the department of surgery, and you doubtless receive ample instruction in relation to it, in the Marine Hospital, by my distinguished colleague, prof. Brainard, yet the cases occurring in private practice are so frequent, and of so much importance, that I shall not hesitate to call your attention frequently to such as may chance to come into my department of this institution.

Judging from what has fallen under my own observation during the last few years, I am led to think that there is very great want of attention to, and accurate knowledge of, the different forms of opthalmia, by the great majority of practitioners of medicine. I fear that very many content themselves with the general diagnosis of "*sore eyes;*" and when they have prescribed blue pill, and epsom salts, internally, with blisters externally, and an eye-water of nitrate of silver, or sulphate of zinc, they have exhausted their store of remedial agents. It seems to be too often forgotten that the eye, with its immediate appendages, includes a large number of distinct anatomical structures, each having its own physiological relations, and amenable to a variety of diseases arising from different causes and leading to equally diverse results. The muciperous glands and follicles in the tarsus of the eye-lids, the conjunctiva, the cornea, the schlerotica, the iris, the ciliary processes, the retina, &c., are all anatomically and physiologically distinct structures, and liable to be separately attacked by disease, just as much as are the pleura, the parenchyma of the lung, and the bronchial mucous membrane. And not only so, but some of these delicate structures are subject to diseases specifically as different from each other, as are acute pneumonia and tubercular phthisis. For proof of this we need only to compare the acute conjunctivitis or common catarrhal opthalmia with the irritable ulcer in the cornea; or rheumatic or syphilitic iritis with that acute and severe conjunctival and corneitic inflammation known by the name of Purulent or Egyptian Opthalmia. The particular cases to which I shall direct your attention this morning, illustrate one of the most important diseases to which the eye is subject, and which is of sufficiently frequent occurrence in practice to command your most careful attention. The patients are these three little girls, all belonging to the Orphan Asylum, and consequently representatives of the poorer classes. Two of them, you notice, are sitting with their faces turned from the light, their chins constantly pressed down upon the sternum, and their eyes firmly closed. I turn them round, I wish you to note their physiognomy, for it is highly expressive and characteristic.

So soon as the light of the window strikes the face, the chin is not only depressed, but the angles of the mouth are drawn down, the eye-brow strongly knit or corrugated, the eye-lids firmly closed, and the face pale, together constituting a position and expression or physiognomy very striking, and seldom seen except in connection with the variety of disease which we have before us. All that is peculiar, however, in the expression of countenance, is derived directly from the extreme sensitiveness of the eyes to the impression of light. This sensitiveness to light is worthy of your attention. It is often so great that any attempt to examine the eye-ball causes streams of tears to flow down over the cheeks, and the most acute suffering to the patient. Little children like the youngest of these will often sit in a dark corner of the room, their faces to the wall, and the chin depressed, and amuse themselves with play-things for hours very cheerfully, while any attempt to bring them to the ordinary light of the windows will be resisted with all the strength they possess.

The same acute sensitiveness causes them when lying down to constantly turn the face to the pillow so long as any light remains in the room. This extreme sensitiveness accompanies the irritable or scrofulous variety of inflammation, whether it is located in the cornea, ciliary processes, iris, or even the follicles and glands in the tarsus of the eye-lids; and is always accompanied by a profuse flow of tears. I am satisfied that this sensitiveness does not depend so much on a morbid condition of the optic nerve or retina as on the sensitive branches of the fifth pair, which are more directly involved in the inflamed textures. This patient, which we shall call Case I., is a girl about 10 years of age. I learn by inquiry that she has had what are usually called "*weak* eyes," for more than a year. They are denominated *weak* because the patient cannot bear without inconvenience exposure to clear sun-light, or to the direct light of a lamp; and because the eye-lids are almost always aglutinated or stuck together on awakening in the morning. If you step forward two or three at a time, and examine closely, you will readily see the nature and extent of the local difficulty.

The tarsus of the lids, especially the upper ones, are thickened, reder than natural, and the outer edge more or less covered with a concrete tenacious substance entangled in the eye-lashes, and which is being constantly secreted by the diseased glands and follicles of the tarsus. There is no redness or appearance of disease in any part of the eye-ball; neither is the conjunctiva lining the eye-lids more than very slightly altered from the natural condition. Hence I may say that the local evidences of disease are limited to the bulbs of the eye-lashes and the follicles contained in the tarsus of the eye-lids. This affection is generally chronic and often of long duration. In many instances it seems to remain nearly stationary for years, occasioning only a swollen and uncleanly appearance of the edges of the eye-lids, and a morbid sensibility of the eye to bright light. But in a majority of cases the morbid changes slowly progress until results of a far more serious character are produced. Slight causes induce frequent exacerbations of inflammation in the glands of the tarsus. This inflammation is very apt to assume the pustular form, constituting what is familiarly denominated the *sty.* The edges of the lids become constantly more thickened, reder, and more destitute of eye-lashes; causing them to look denuded.

The bulbs of the lashes being involved in the disease, such of the latter as do not fall out, grow very irregular and imperfect. The edges of the lids sometimes become everted, constituting the *Ectropion* of your books; more frequently they are inverted (*Entropion*), and the stunted, irregular eye-lashes are brought more or less in contact with the cornea, irritating it and inducing sooner or later partial opacity with dimness of vision. Such are the general tendencies and results of this apparently simple variety of disease. In the case before us it is yet in its early stage, there being no loss of eye-lashes or mechanical irritation of the cornea. The disease is met with at all periods of life, though more frequently in childhood.

Two classes of persons are peculiarly liable to it, namely, the scrofulous and the intemperate. You will readily infer from the age, sex, and delicacy of features presented by this patient, that she belongs distinctly to the former class. Of the treatment I shall speak after examining the remaining cases.

Case II. This patient is a little girl aged only four years. As I raise her chin from the breast and bring her face fully into view, you see her eyes firmly closed, the eye-brows knit, the edges of the lids red and swollen, and an abundant muco-purulent matter running from the inner angle over the cheeks. As you approach and examine more minutely, you see the lower lid of the left eye much excoriated and inflamed on its outer surface, and as I open the lids you see what may appear at first to be a small whitish or opaque spot on the cornea a little above the center. On more careful examination, while the eye-ball turns in different directions, you see that spot to be a simple excavated ulcer; and that on the schlerotic coat there is a zone or belt of intense redness, one or two lines in width, immediately encircling the margin of the upper half of the cornea. A few red vessels are obscurely seen penetrating a little way into the cornea, while on the other side of the red zone, the redness rapidly diminishes as we recede from the cornea. You find here no general conjunctival redness, thickening, or granulation. The right eye of this patient is affected in precisely the same manner as the left, though less severely, and the cornea not yet presenting any appearances of ulceration. In this patient, then, we have the same affection of the tarsus of the eye-lids as in Case I. though in a more acute form; and in addition a very severe and important ulcerative inflammation of the cornea.

Case III. This patient, a girl aged about seven years, presents you the same striking physiognomy as the one just examined. There is the same corrugation of the eye-brow, firm closure of the eye-lids, depression of the angles of the mouth, and persistent tendency to hold the face downward. But you see here no affection of the tarsus of the eye-lids. The latter are healthy both without and within. But as I force open the lids, you see here a condition of the cornea very similar to that in Case II. There is an excavated ulcer of considerable size directly over the pupil, another smaller one near the upper and outer margin of the cornea, and the same zone or belt of redness on the schlerotic coat encircling in this case the entire base of the cornea. The cornea of the other eye is affected in a similar manner; but as all exposures to the direct light are very painful, causing as you see here the most profuse flow of tears, we will not ask you to examine it. Having each of you carefully examined these cases, I desire now to impress upon your minds some of their characteristic or diagnostic features, their tendencies, and final results. You will then easily comprehend the appropriate treatment. A moment's reflection will show you that the most characteristic symptoms are, the position and physiognomy; the careful avoidance of light; the profuse flow of tears, mingled with only a small proportion of mucus; the location and limited extent of the redness, with more or less alteration in the cornea; and the chronic or protracted continuance of the disease. From ordinary catarrhal opthalmia it differs in almost every particular. In the latter the whole conjuctiva is red and more or less swollen, instead of presenting a limited zone of redness immediately around the base or circumference of the cornea as in these cases. Instead of great intolerance of light with profuse flow of tears, conjunctivitis is accompanied by a simple increased sensibility, a feeling of heat and roughness as though some foreign substance was in the eye, and after the first two or three days, a moderate discharge of muco-purulent matter; and the cornea, if affected at all, only becomes so secondarily after the conjunctiva lining the eye-lids has become thick and granular. From that severe and destructive form of inflammation termed Egyptian or Purulent Opthalmia, the cases before us differ still more widely. The former always commences suddenly, and progresses so rapidly that in four days the conjunctiva and eye-lids are swollen to such an extent as to entirely close the eye and fill up the circumference of the orbit, and is accompanied by great pain and symptomatic fever. The discharge too, after the two first days, is copious and almost entirely purulent.

None of these symptoms mark the early stage of the cases you have been examining. These three cases belong to the class of Irritable or Scrofulous inflammations; the first being limited to the tarsus of the eye-lids, the second involving the tarsi and cornea both, and the third the cornea alone. Like most other scrofulous affections, they generally commence insidiously, progress slowly and persistently, and if uncontrolled by proper treatment, generally end in alteration or destruction of the tissue affected. The changes wrought by the disease in the tarsus of the lids, I have already detailed. Located in the cornea, as in Cases II. and III., the constant tendency is to a slow ulceration, by which the texture of the cornea is destroyed in one or more places. Commencing on the surface by what appears to be a small whitish spot, often not half as large as a pin's head, the ulcer gradually extends both in circumference and depth, until it sometimes penetrates all the layers of the cornea and allows the delicate lining of the anterior chamber to protrude. Examined carelessly, these ulcers are not unfrequently regarded as mere *films* or partial opacities on the surface of the cornea, an error of much practical importance; and one that may be easily avoided by examining carefully while the eye-ball is allowed to move in different directions so as to reflect the rays of light at different angles to the eye of the examiner. Properly examined, they will always be found, however small, to be excavated, that is, accompanied by positive loss of substance. And often there is so little deposite of opaque matter in the diseased part, that the ulcer itself remains a long time nearly transparent.

The prognosis in the variety of Opthalmia illustrated by these cases is generally favorable. For though slow in its progress, often persisting for many months, the disease will almost always yield to appropriate treatment. Occasionally, however, the cicutrix left after the ulcer is healed, is so large and opaque as to seriously obstruct the entrance of light into the pupil of the eye, and vision consequently remains very imperfect. The indications for treatment in this variety of disease cannot be studied too carefully, for I think they are more frequently mistaken than in any other form of Opthalmia.

I have more than once seen patients of this class with pale faces, soft flaccid muscles, and all the marks of positive anemia or deficient nutrition, subjected to the influences of dark rooms, blue pills, epsom salts, and blisters; while to the extremely sensitive eyes were applied daily, weak solutions of nitrate of silver, sulphate of zinc, &c., and sometimes the solid caustic to the ulcers. So far as I have been able to observe, such treatment is not only founded on wrong ideas in relation to the nature of the disease and the general condition of the patient, but is positively injurious in its effects.

The truth is, that the whole class of scrofulous, irritable, or ulcerative inflammations of the cornea, of which the cases before you are good representatives, are connected with and dependent on, a debilitated and depraved condition of the system. And the first and most important indication for treatment is to correct this general pathalogical condition, by means which will improve functional action, increase the tone of the solids, and improve the quality of the fluids. The second indication is to allay the morbid sensitiveness of the eye, and excite a more healthy reparative action in the parts affected with local disease. To accomplish the first, we must rely chiefly on a nutritious diet and the judicious use of tonic and alterative remedies. The best articles of food are milk, tenderly cooked meat, light stale bread, potatoes, rice, and ripe fruits. The best drink is water, though tea, coffee, and chocolate may be allowed very moderately. During the last ten years I have seen and treated many such cases as we have before us this morning; and of all the internal remedies I have used, none have so uniformly and promptly arrested the progress of the disease as the union of the bi-chloride of mercury in small doses with the cinchenæ. And I will prescribe for these children, this morning, as follows:

℞. Bi-Chloride Hydrarg. - - 1 gr.

Tinct. Cinchonæ, - - - ℥ii.

Mix, and give the youngest 20 drops in a table spoonful of sweetened water three times a day. To the two older ones give 30 drops in the same manner. At the same time, in fulfilment of the second indication, I will direct for local application the following, viz:

℞. Sulph. Morph. - - - 4 grs.
Water, - - - - - ℥i.

Mix, and drop two or three drops into each eye three or four times a day. Also,

℞. Ammoniated Hydrarg. - - 15 gr.
Simple Cerate, - - - ℥ss.

Mix very thoroughly, and apply it to the edges of the eye-lids from one angle to the other, every night before the patient goes to sleep.

These remedies will probably need to be continued with very little change from ten to twenty days. The patients will not get well in that time, but you will be able to notice important changes, which will often indicate the necessity of changing the remedial agents. In the length of time I have mentioned, we generally find the sensitiveness of the eye much diminished, the tears flow much less freely, the red zone around the cornea is less intense, and the ulcers have visibly diminished in size. If, as is often the case, the patients exhibit also an increase of strength, flesh, and cheerfulness, with a regular and healthful condition of the alimentary canal, no change need be made except to diminish the doses of the bi-chloride and tinct. of cinchonæ, first to twice a day and subsequently to once, at which rate it may be continued until the patients are quite well. In many cases, however, while the improvements which I have mentioned take place in the eyes, the medicine induces a slight irritating effect on the mucous surfaces, characterized by occasional griping pains in the abdomen, a slightly redened and tender appearance of the edges of the tongue and sometimes of the whole mouth, peevishness in children, and feelings of languor and discomfort in the adult. When such symptoms occur during the use of the mercurial preparations they should never be disregarded, but the remedy should be immediately discontinued and something more tonic and less liable to irritate the mucous membranes substituted in its place. For this purpose I have generally resorted to one or the other of the following formulæ:

℞. Glycerine, - - - - ℥ii.

Syrup of Iodide of Iron, - ʒiii.

Mix, give at each meal-time from 20 to 60 drops, according to the age of the patient; or,

℞ Tinct. Cinchonæ, - - - ℥ii.

Iodide of Potassa, - - ʒi.

Mix, dose the same as the preceding; or,

℞. Cod Liver Oil, - - - ℥jss.

Syrup of Iodide of Iron, - - ʒiii.

Pulv. G. Arabac, } aa ʒiii.

White Sugar, }

Rub together and add Mint Water, ℥jss.

Mix, and give from ʒss. to ʒii. three times a day, always shaking the vial before the dose is poured out.

Either of these preparations will generally produce the desired effect, by improving rapidly the nutritive functions and general health of the patients; and often nothing else is required but a continuance of the same local remedies as before and the cure will be completed in from three to six weeks. In some patients, however, the scrofulous constitution is more strongly developed, and the treatment must be continued several months. In such I have found it of great advantage to return to the use of the bi-chloride of mercury and tincture of cinchonæ every two or three weeks, continuing it each time from six to ten days.

You will occasionally meet with patients who cannot take the bi-chloride of mercury in even so minute doses without inducing undue irritation of the mucous membrane of the alimentary canal. In such cases I have found the best substitute to consist of proto-iodide of mercury in doses of one-eighth of a grain to one grain, according to the age of the patient, and combined with Dover's Powder sufficient to produce a gentle anodyne influence.

In very debilitated subjects I have added to this powder from ¼ to 1gr. of sulphate of quinine or sulphate of cinchonæ.

While using the proto-iodide of mercury the patient must be watched quite as carefully as though the bi-chloride was the remedy given, and it should always be interrupted or discontinued before the slightest symptoms of salivation are induced.

For local application to this class of sore eyes, I have only mentioned the solution of morphine to the eye-ball and a mild mercurial ointment to the tarsus of the lids. For the morphine, however, we may substitute any of the strong norcotic vegetable alkloids, such as conia, atropia, &c.; the object being to directly lessen the irritability of the textures and the extreme morbid sensitiveness of the nervous filliments connected with them. But, gentlemen, the present clinque hour has expired, and I cannot detain you longer this morning.

You will have an opportunity of seeing these cases again in connection with three or four others, after a few days, when some other points relative to the treatment will be more fully discussed.

